# The role of semantic processing in reading Japanese orthographies: an investigation using a script-switch paradigm

**DOI:** 10.1007/s11145-017-9796-3

**Published:** 2017-11-08

**Authors:** Alexandra S. Dylman, Mariko Kikutani

**Affiliations:** 1 Department of Psychology, Mid Sweden University, 831 25 Östersund, Sweden; 2 Faculty of Psychology, Doshisha University, Kyoto, Japan

**Keywords:** Japanese reading, Script switching, Reading aloud, Semantic decision, Japanese orthographies

## Abstract

Research on Japanese reading has generally indicated that processing of the logographic script Kanji primarily involves whole-word lexical processing and follows a semantics-to-phonology route, while the two phonological scripts Hiragana and Katakana (collectively called Kana) are processed via a sub-lexical route, and more in a phonology-to-semantics manner. Therefore, switching between the two scripts often involves switching between two reading processes, which results in a delayed response for the second script (a script switch cost). In the present study, participants responded to pairs of words that were written either in the same orthography (within-script), or in two different Japanese orthographies (cross-script), switching either between Kanji and Hiragana, or between Katakana and Hiragana. They were asked to read the words aloud (Experiments 1 and 3) and to make a semantic decision about them (Experiments 2 and 4). In contrast to initial predictions, a clear switch cost was observed when participants switched between the two Kana scripts, while script switch costs were less consistent when participants switched between Kanji and Hiragana. This indicates that there are distinct processes involved in reading of the two types of Kana, where Hiragana reading appears to bear some similarities to Kanji processing. This suggests that the role of semantic processing in Hiragana (but not Katakana) reading is more prominent than previously thought and thus, Hiragana is not likely to be processed strictly phonologically.

**Electronic supplementary material:**

The online version of this article (10.1007/s11145-017-9796-3) contains supplementary material, which is available to authorized users.

## Introduction

While the vast majority of orthographies use either a logographic script where characters represent units of meaning (such as in Chinese), or a phonological script where characters represent units of sound (such as alphabetic orthographies), Japanese uses both these systems simultaneously and in combination. Specifically, Japanese uses the *logographic* (or *morphographic*) script Kanji, as well as the two *phonological* scripts Hiragana and Katakana (collectively called Kana). Kanji (originally derived from Chinese characters) is commonly used for writing content words—most nouns, verbs and adjectives are written in Kanji—and Kanji characters, alone or in combination with other characters, represent whole words. In contrast, Kana has clear and direct character-to-sound correspondences where each Kana represents a Japanese mora (the elementary syllabic units in Japanese speech, found to be the smallest processing unit in Japanese language processing; e.g., Ida, Nakayama, & Lupker, 2015; Kureta, Fushimi, & Tatsumi, 2006; Tamaoka, 2014). Katakana is typically used to write foreign loanwords, and may also be used for emphasis (in place of italics in, for example, English). The purpose of Hiragana is more varied and its uses range from writing function words to forming complete words by being combined with Kanji characters (for a more extensive description of the written Japanese language, see Kess & Miyamoto, 1999, or Tamaoka, 2014).

Early research on reading Japanese suggested that processing of Kanji follows a strict semantics-to-phonology route, while Kana is processed in a phonology-to-semantics manner (e.g., Feldman & Turvey, 1980; Goryo, 1987; Kimura, 1984; Morton & Sasanuma, 1984; Nomura, 1981; Sasanuma, 1975; Sasanuma & Fujimura, 1971; Shimura, 1987). Within a framework of a dual-route model of reading (e.g., Coltheart, 1978, 2005; Coltheart, Curtis, Atkins, & Haller, 1993; Coltheart, Rastle, Perry, Langdon, & Ziegler, 2001) this would suggest that Kanji words are read via a semantic-lexical route, while Kana words are read via the non-lexical phonological assembly route. As Wydell and Kondo (2015, p. 240) point out, reading Kanji “may require a greater weighting for the whole-word level contribution in the computation of phonology from orthography, as the relationship between orthography (Kanji) and phonology (pronunciation) is opaque”. Furthermore, the use of Kanji helps disambiguate between the large number of homophones in the Japanese language (in part due to the comparatively limited number of morae in the Japanese language, e.g., Kess & Miyamoto, 1999; Kinoshita & Saito, 1992), and so, it is thought that Kanji has stronger orthography-to-semantic links. In contrast, Kana characters map more directly onto their corresponding phonology and thus have more direct orthography-to-phonology connections (e.g., Feldman & Turvey, 1980; Goryo, 1987; Morton & Sasanuma, 1984; Saito, 1981). According to this view, reading Kanji and Kana use two distinct routes and readers switch between these two.

The switching of reading processes is widely researched in the bilingual literature using the switching paradigm, in which bilingual participants read out pairs of words either written in the same language (for example both English) or in different languages (for example one in German and the other in English) consecutively. Studies using this paradigm often observe a so called switch cost effect, which is signified by a delay in producing speech when bilinguals switch between their two languages (e.g., Dalrymple-Alford, 1967; Macnamara & Kushnir, 1971, Meuter & Allport, 1999; Peeters, Runnqvist, Bertrand, & Grainger, 2014; Thomas & Allport, 2000).

Shafiullah and Monsell (1999) adopted this switching paradigm to investigate script switch costs (i.e., a delay in reaction times when switching from one script to another, compared to within-script trials) both for reading and semantic categorization in Japanese. In a series of experiments, manipulating a number of characteristics of the stimuli (such as visual complexity, spatial extent and number of characters), they found a significant delay in reading time when participants switched between Kanji and Kana compared to when they read two words written in the same script successively. Importantly, the magnitude of the switch cost was equivalent for switching from Kanji to Kana and from Kana to Kanji despite Kanji being the more familiar form, and this finding was replicated when participants semantically categorized each word instead of reading them aloud.

Shafiullah and Monsell (1999) concluded that the presence of the switch costs indicates the non-overlapped representations, mechanisms or pathways used to decode Kanji and Kana scripts, and dismissed other possibilities, including a familiarity effect. The familiarity effect is signified by faster reading times for Japanese words written in the usual, or familiar, script as compared to transcriptions of Kanji or Kana words written in an unfamiliar script, suggesting preferential processing for words written in the familiar script (e.g., Besner & Hildebrandt, 1987; Chikamatsu, 2006; Tamaoka, 1997). When a reader is presented with a pair of words typically written in Kanji in a successive manner, but one is written in the unfamiliar Hiragana form and the other is in the familiar Kanji form, decoding two words with unbalanced familiarity alone may cause the switch cost. Specifically, it may be expected that switching from Kanji to Hiragana (from familiar to unfamiliar) takes longer than switching from Hiragana to Kanji (from unfamiliar to familiar) because seeing an unfamiliar form of a word after decoding the familiar form may surprise the reader. However, this was not found by Shafiullah and Monsell (1999) in their Experiment 5, for which they used a word set with equivalent familiarity for words written in Hiragana and Kanji. However, we would like to point that this result is insufficient to dismiss the possibility that familiarity alone can induce the switch cost as switching between Kanji and Hiragana always involves switching the reading pathways. In order to isolate the familiarity effect, two different scripts engaging the same reading pathway should be compared, and the results contrasted to the Kanji–Hiragana switch. To examine the impact of script familiarity, the present research used words generally written in Kanji for a Kanji–Hiragana switch task while using words normally written in Katakana for a Katakana–Hiragana switch task, ensuring that Hiragana always served as transcriptions of the familiar form of the word. If script familiarity alone can induce the switch cost, it should be observed when switching between Katakana and Hiragana. Furthermore, the two Kana scripts are more visually similar than Kanji and Kana and so we are able to prevent potential confounds of visual properties. Even if the familiarity effect is not solely responsible for the switch cost, it may manifest in addition to the switch cost caused by the shift of reading processes. The present study investigates the effect of script familiarity by comparing Kanji–Hiragana switch and Katakana–Hiragana switch.

Indeed, Shafiulla and Monsell (1999) investigated a Katakana–Hiragana switch and did not find any significant switch cost. Hiragana and Katakana share numerous characteristics such as structure and segmentation, age of acquisition (both scripts are learnt in parallel in school), and configuration (the two systems have the same number of orthographic units so that for each Hiragana there is a corresponding Katakana character and vice versa). Some studies have found no behavioural differences between Hiragana and Katakana in experimental tasks (e.g., Shafiulla & Monsell, 1999; Tamaoka; 1997) lending further support for the assumption that readers use highly similar processing mechanisms. Hiragana and Katakana have, therefore, often been used interchangeably in experimental settings (e.g., Feldman & Turvey, 1980). However, we would like to argue that there are important, fundamental differences in terms of intrinsic purpose and function of each script in Japanese writing. For example, Kambe (1986) found that participants focus more on words containing Kanji or Katakana (normally used to write content words), than words consisting solely of Hiragana characters (which are usually used to write function words). As mentioned, the Japanese language contains a large number of homophones, which are indistinguishable when written in Hiragana, and the use of Kanji helps disambiguate between them. However, Katakana words, by default, contain fewer homophones as Katakana tends to be used to write foreign loanwords imported from languages with a larger set of phonemes than Japanese (which naturally results in fewer homophones). The speed of Kana processing is also found to vary depending on the function of the words. For example, Tamaoka (1997) observed no significant difference in response time between reading Hiragana and Katakana when both of these were transcriptions of Kanji words,^1^ while Besner and Hildebrandt (1987) found that real Kana words were read significantly faster than Kana transcriptions of Kanji words. Considering the function of the words, the Katakana–Hiragana switch experiment by Shafiulla and Monsell (1999) contains a significant methodological concern. On the one hand, they used foreign loanwords normally written in Katakana for the Katakana words, and indigenous Japanese words normally written with infrequent and obscure Kanji forms as the Hiragana words for Katakana–Hiragana switch. On the other hand, they used Kanji words and their Kana transcriptions for their Kanji-Kana switch experiment. This methodology is problematic because of the inconsistency of the function of the Hiragana words used (i.e., real words in some of the experiments and transcriptions of Kanji words in others). For a clear comparison between the Kanji–Hiragana switch and the Katakana–Hiragana switch the function of the script should be consistent. The present study controlled for this aspect.

Apart from the pathway switching and word familiarity, the visual property of the different scripts could also induce the switch effect. Shafiulla and Monsell (1999) pointed out that the size of the “attentional spotlight” influences Kanji and Kana decoding differently. The attentional spotlight is an area within the entire visual field where the attention is most focused, and we need to constantly alter the size of the spotlight in accordance with the size of stimuli. Since a larger number of Kana is often required to represent the same word written in Kanji, the adjustment of the attentional spotlight is likely to differ between the Kana and Kanji decoding, and this could cause the switch effect. By using words in altered font sizes, Shafiulla and Monsell (1999) observed some support that the size of the attentional spotlight is responsible for the switch cost, and thus opened the possibility that the cost can manifest without any switching of the reading processes. In order to dismiss this visual property account, we need to ensure that participants are actually engaging in two different reading pathways for Kanji and Kana. Since Kanji is processed in a more direct orthography-to-semantics manner, it is thought to gain faster access to semantic meaning than Kana. On the contrary, reading Kanji is harder than reading Kana because Kanji characters typically have at least two possible pronunciations, *Kun*-*reading* (the original Japanese name), and *On*-*reading* (derived from the Chinese names that came with the importation of the Chinese characters into the written Japanese language). This makes reading Kanji words inherently effortful as Japanese readers must choose between several possible phonological forms before proceeding to produce the intended word in speech (e.g., Verdonschot, La Heij, Paolieri, Zhang, & Schiller, 2011; Verdonschot et al., 2013). These characteristics lead us to assume that semantic categorization of Kanji words can be completed more quickly than that of Kana words. Surprisingly, however, this was not the case when Shafiulla and Monsell (1999) investigated Kanji-Katakana switch using a semantic categorization task (Experiment 6), to which they commented that “given the widespread assumption that the mapping of Kana to meaning is less direct than for Kanji, it is of some interest that semantic categorisation performance was so similar for the Kanji and Katakana words” (p. 593). As the robust switch cost was present for this experiment it can be taken as further support that the switch cost can occur without the switching of the reading pathways. Furthermore, this could indicate that Kana is not processed purely in a phonology-to-semantic manner and the weight of the semantic processing of Kana words might be more significant than it is initially thought. Indeed, some researchers argue that the processing mechanisms of the different orthographies are complex and overlapping, with studies showing lexical aspects for Kana reading (Besner & Hildebrandt, 1987).

In order to examine whether Kana and Kanji reading engage in different pathways during the script switch paradigm, the present study used paired words that were either semantically related or unrelated. When two related words are read aloud, the processing of the second word is likely to be aided by the first word, particularly for Kanji reading where semantic processing should be completed quickly and before access to phonological representations. The semantic relatedness of the pair, however, may not impact Hiragana reading to the same extent, as reading in this phonologically transparent script does not need to rely on semantic processing as much as does Kanji reading. If Kanji and Hiragana reading actually engage in different pathways, the overall response time for Kanji reading should be affected more by the semantic relatedness of the word pairs than Hiragana reading regardless of the script switch. We also employed a semantic decision task in which participants responded whether the paired words belonged to the same semantic category or not. This task was used because the benefit of word relatedness on Kanji processing (working in a stricter semantics-to-phonology fashion) may be compromised by a general difficulty in reading Kanji aloud compared to reading Kana. If the impact of a semantic effect is equivalent for Kana and Kanji, we cannot conclude that reading the two types of scripts engages in two systems, at least in the present tasks.

In our studies, participants read aloud (or made semantic decisions about) pairs of words, which were either written in the same script (non-switch trials) or in different scripts (switch trials), and the time taken to respond to the second word was measured. For Kanji–Hiragana experiments (Experiments 1 and 2) we used words that are normally written in Kanji and their Hiragana transcriptions. In order to make parallel comparisons to these, Katakana–Hiragana experiments (Experiments 3 and 4) used foreign loanwords normally written in Katakana for the Katakana words and their transcription as Hiragana words. The detailed predictions for following experiments are laid out in each section.

## Experiment 1: Kanji–Hiragana reading

This experiment aimed to examine whether the script switch cost between Kanji and Hiragana observed by Shafiulla and Monsell (1999) is replicated and, more importantly, whether the magnitude of the semantic facilitation differs between the two scripts. Participants were asked to read out pairs of words, which were either written in the same script (non-switch trials) or in different scripts (switch trials). Based on Shafiulla and Monsell (1999) findings, we expected to observe a basic script switching cost where non-switch trials are faster than switch trials, for both Kanji and Hiragana. All the words used in the experiment are normally written in Kanji and so, an asymmetric script switch cost signified by a larger delay for the switch from Kanji to Hiragana (from familiar to unfamiliar) over the switch from Hiragana to Kanji (from unfamiliar to familiar) might be found due to the familiarity effect. In addition, the stronger orthography-to-semantics links for Kanji reading is expected to lead to faster reading times for related compared to unrelated Kanji trials while it may not be the case for Hiragana words if they are processed purely phonologically.

### Method

#### Participants

Twenty-eight native Japanese speakers (18 women and 10 men) living in Japan participated. Their mean age was 19.6 years (SD = .95 years), and none of them reported having any reading difficulties. They did not participate in any of the other experiments reported in the present study.

#### Stimulus materials

Eighty Kanji words were selected to create 40 semantically related pairs (see Table 4 of “Appendix”). The two words in each pair belonged to the same semantic category on the same basic-level (e.g. ‘summer’ and ‘winter’, or ‘dog’ and ‘cat’), avoiding associates (e.g., ‘dog’ and ‘bone’) or other types of relationships such as different-level pairs (e.g., ‘flower’ and ‘tulip’), whole-parts (e.g., ‘car’ and ‘engine’) or associates from different grammatical classes (e.g., ‘bed’ and ‘sleep’). Half of the 40 pairs consisted of single-character words and the other half consisted of two-character words. Four additional pairs (two each for the single- and two-character word pairings) were created for practice trials. The 40 experimental pairs would consist of a total of 120 Kanji characters if each character appear only once (some characters appeared more than once as a composite of different words). Two characters used as single-character words were also a part of two different two-character words. A total of four characters used as a composite of two-character words appeared twice each in the set of twenty pairs. This resulted in 114 Kanji characters to be used in the present experiment. All the selected Kanji were familiar to adult readers and were taken from the list of “regularly-used Kanji characters” called the Jouyou list issued by Japanese Ministry of Education in 1981. Japanese speakers normally learn most of the characters on this list between the ages of 6–15 during which they enrol in compulsory education. Although the age of acquisition for each character varies, 105 out of 114 are learned between the age of 6–12 at primary education level, and only 9 are learned at the secondly level education.

The single-character words had two to three morae when read in *Kun*-*reading* (the original Japanese name). Although they could be read differently when applying *On*-*reading* (derived from the Chinese names), Japanese readers tend to use *Kun*-*reading* when a single-character is presented alone (Wydell, 1998), and so we regarded the *Kun*-*reading* as correct answers. The majority of the two-character words were Kango (words of Chinese origin) and thus *On*-*reading* is applied and there is no ambiguity in how they should be read. None of the paired words were phonologically related to each other. No visual component was shared among the Kanji characters in the same pair, so the paired Kanji words were visually dissimilar to each other.

Forty semantically unrelated pairs were created by altering the word pairings of the semantically related list (see Table 5 of “Appendix”). These semantically related and unrelated pairs were called “related Kanji non-switch” and “unrelated Kanji non-switch” lists respectively. Similar lists were created with Hiragana transcripts of the Kanji words to form “related Hiragana non-switch” and “unrelated Hiragana non-switch” lists. The number of Hiragana characters in each word ranged from two to five. When written in Hiragana, the semantics of some words could be ambiguous due to existing homophones. Six out of the 40 single-character words and one in the two-character word set have potential homophones, and the manipulation of semantic relatedness for the pair consisting of these words could be insufficient. Avoiding homophones completely was difficult because they were common, which is why we decided to leave these pairs in the experimental set and paid particular attention to them during the analysis.

For the semantically related and unrelated Kanji switch lists, the first word was written in Hiragana and the second word was written in Kanji. The order of the scripts was reversed for the Hiragana switch lists. These eight lists gave the experiment a 2 × 2 × 2 design with the factors of relatedness (semantically related vs. unrelated), script (Kanji vs. Hiragana) and switch (switch vs. non-switch). Eight additional pairs representing the eight conditions were created for practice trials. Ensuring that words from two consecutive trials were phonologically, semantically and orthographically dissimilar, we ended up with eight sets of pseudo-randomised trial orders without showing the same word pairing consecutively. The presentation orders of the paired words were counterbalanced across the participants so that half the participants responded to the pairs of words in one order (A–B) while the other half responded to the pairs of words in the reversed order (B–A). The Kanji and Hiragana words were typed in black MS Gothic font. The size of each character was to fit in 4.5 cm^2^ when presented on the computer screen. A 5 cm × 20 cm rectangle filled with visual noise was used as a visual mask presented between words.

#### Procedure

The participants were tested individually on a MacBook Air laptop (13” screen) using the software program SuperLab version 5. The words were presented on the screen one at a time and participants were asked to read them aloud as quickly and as accurately as possible. The experiment consisted of 8 lists with 40 word pairs totalling 320 trials, and participant read two words per trial to make a total of 640 responses.

The word pairs were presented successively in each experimental trial. Initially, the first word was presented on the screen until the participant produced a vocal response, which was detected by an Elecom usb-headset connected to SuperLab’s voice key. Once the voice key was triggered, the word was replaced by a visual mask for 1 s, which was followed by the second word in the pair. The same visual mask was used in the inter-trial intervals to remove any obvious indication of the start of each separate trial. This was to prevent participants from memorizing the pairings of the words. Participants started the experiment with eight practice trials, and they were allowed to take a short break after every 80 trials. The experimenter recorded response accuracies for each trial.

### Results and discussion

Responses for the second word of each pair were analysed. Responses that were incorrect, hesitant or non-verbal, or failed to trigger the voice key were removed. In order to clean up the data further we excluded responses that fell outside of 2.5 standard deviations from each participants’ mean in each condition. Removed responses were only 1.7% of the total number of responses, and so participants were very accurate in reading the present words aloud. Preliminary analyses conducted separately for the single- and two-character words, and with or without the pairs with possible homophones found no impact of them, so these factors were collapsed. Means of non-excluded responses were calculated for each participant in each condition and are reported in Table 1.

**Table 1:** Results from Experiment 1, Kanji–Hiragana reading

	Kanji		Hiragana	
	*M*	*SD*	*M*	*SD*
*Related*				
Non-switch	664	55	618	42
Switch	634	62	626	55
Switch cost =	− 30		8	
*Unrelated*				
Non-switch	663	49	624	47
Switch	647	53	640	46
Switch cost =	− 16		16	

Mean response times (in milliseconds) and standard deviations, for Kanji and Hiragana words at each trial type. Switch-cost = Switch trials minus non-switch trials

The data were analysed (separately for participants and for items) using three-way related ANOVAs^2^ with the factors semantic relatedness (related vs. unrelated), script (Kanji vs. Hiragana), and switch (switch vs. non-switch).

The three-way related ANOVA found a main effect of relatedness, *F*_1_(1, 27) = 16.94, *MSe* = 221, *p* < .001, η_p_^2^ = .39; *F*_2_(1, 78) = 7.73, *MSe* = 1667, *p* = .007, η_p_^2^ = .09; RTs were faster when the target words were semantically related to the preceding word than when they were unrelated (635 vs 644 ms). The main effect of script was also significant, *F*_1_(1, 27) = 28.07, *MSe* = 1261, *p* < .001, η_p_^2^ = .51; *F*_2_(1, 78) = 68.19, *MSe* = 1534, *p* < .001, η_p_^2^ = .47; RTs for reading Hiragana words were faster than for Kanji words (627 vs. 652 ms). A significant two way interaction between script and switch was found, *F*_1_(1, 27) = 16.78, *MSe* = 1035, *p* < .001, η_p_^2^ = .38; *F*_2_(1, 78) = 26.67, *MSe* = 1481, *p* < .001, η_p_^2^ = .26. Simple main effects showed a switch cost for Hiragana words with faster RTs for the non-switch than for switch trials (620 vs. 633 ms, *p* = .011), while the reverse was true for Kanji words (non-switch trials = 664 vs. switch trials = 640 ms, *p* < .001). No other effects or interactions were significant both by participants and by[items]. A three-way analysis of variance performed on the proportions of errors and excluded responses found no significant effects.

Generally, reading times were faster for Hiragana than Kanji trials, which corresponds to previous findings of slower Kanji reading compared with Kana reading (e.g., Besner & Hildebrandt, 1987), and particularly the general processing cost associated with reading aloud Kanji words, due to the multiple pronunciations of Kanji characters (e.g., Verdonschot et al., 2011, 2013), and also supports the notion that Kanji words are read in a semantics-to-phonology manner.

Furthermore, a reliable switch cost was found for the Hiragana trials, meaning that participants were faster at naming a Hiragana word when the previous word was also written in Hiragana than when the previous word was written in Kanji. Surprisingly, however, the current study found no switch cost in the other direction, that is, when switching from Hiragana to Kanji. In fact, non-switch Kanji–Kanji trials were *slower* than Hiragana–Kanji trials. The present results are thus noticeably different from the findings of Shafiullah and Monsell’s (1999) who found switch costs of equivalent magnitude for both switching directions. Instead, the results imply that the switch effect is caused by the general difficulty in reading Kanji aloud. It was consistently found that reading the second word of the pair (either written in Kanji or Hiragana) was slowed down when the first word was written in Kanji, indicating that there were some after-effects of processing the initial Kanji word which influenced the reading time for the second word. This was not found by Shafiullah and Monsell (1999), and it may be because all of their experiments employed “pure blocks” in which the word stimuli used the experiments were shown in either of the scripts without switching. Each pure block contained 30 trials and several pure blocks were interspersed with mixed blocks which consisted of switch and nonswitch trials. In this case, the Kanji pure blocks could have served as practice for their participants to be familiarized with reading multiple Kanji words (which were also used in the mixed blocks) consecutively. Consequently, the participants might have been able to read the Kanji words relatively effortlessly overall. The impact of the general difficulty in reading Kanji words in their study, therefore, could have been reduced compared that of the present study, and this may explain why the bidirectional switch effect was observed by Shafiullah and Monsell (1999) but not in the current experiment.

The present results do not provide strong evidence that the changing of reading pathways occurred during the task, and this is partly supported by the impact of semantic relatedness. The faster responses for semantically related pairs compared to unrelated pairs indicate semantic facilitation. Importantly, the absence of the relatedness by script interaction means that the facilitation was observed not only for Kanji reading but also for Hiragana reading. This implies that semantic processing is not only involved but more important in reading words written in Hiragana than previously thought. Although the interaction was absent, the impact of semantics appears to be slightly weaker for Kanji trials especially when there was no script switch. This is puzzling because Kanji non-switch trials were expected to gain most benefit from the semantic relatedness. We assume that this may be related to the general difficulty in reading Kanji aloud in the present task rather than an insufficient manipulation of word pairs as the main effect of relatedness was clearly present in the analysis. In order to confirm this, the next experiment used a semantic decision task with key-press responses. This task was used in order to test the semantic priming effect in a context where participants do not need to pronounce the Kanji words aloud, thus removing the issue of selecting the correct phonological response.

## Experiment 2: Kanji-Hiragana semantic decision

The purpose of Experiment 2 was to examine semantic processing involved when participants were not required to produce the words aloud. To this end, Experiment 2 used the same set of stimuli as in Experiment 1, but instead of reading the words, participants were asked to decide whether the two words in each word pair belonged to the same semantic category. Since this task does not involve any speech production, the difficulty in responding to Kanji and Hiragana words are more equivalent compared to the reading task, and thus we should be able to observe the extent of switch cost. It is also important to examine whether the Hiragana processing is engaging the phonology-to-semantic pathway. This processing style should take longer to reach a correct answer in this task compared to the semantic-to-phonology pathway, and so overall response times for Hiragana words should be slower than those for Kanji.

### Method

#### Participants

Twenty-eight native Japanese speakers (21 women and 7 men) living in Japan participated in Experiment 2. Their mean age was 19.2 years (SD = .72 years). None of the participants reported having any reading difficulties. They did not participate in any of the other experiments in the present research.

#### Stimulus materials

The word lists created for Experiment 1 were used in this experiment.

#### Procedure

The participants were tested individually on a Dell inspiron laptop (15” screen) using the software program E-Prime version 2.0. In each trial a pair of words were presented successively. The trial started with a 500 ms fixation cross at the centre of the screen, which was followed by the first word of the pair presented for 1 s. Next, a visual mask appeared on the screen for 1 s and then the second word was shown until a response was made. Participants were asked to judge whether the two words were semantically related or not by pressing the allocated key on the keyboard. The inter-trial interval was a blank screen for 500 ms. Identical to Experiment 1, there were switch and non-switch as well as semantically related and unrelated pairs for Kanji and Hiragana, which resulted in eight conditions with 40 word pairs each. The total of 320 trials were randomly divided into four blocks. The order of the blocks, and of the trials within each block, was randomized across participants. The presentation order of the words for each pair and the location of the response keys on the keyboard were also counterbalanced.

### Results and discussion

Reaction times were measured for deciding whether the second word in each pair was semantically related or unrelated to the first word in the pair. Incorrect responses were excluded from the analyses of the reaction times. Additionally, responses outside 2.5 standard deviations from each participants’ mean for each condition were excluded, following which means of non-excluded responses were calculated for each participant in each condition. Due to semantic facilitation, it is expected that responses for related trials are made generally more quickly than those for unrelated trials, and so, the responses for the two types of trials were treated separately. Table 2 shows the mean reaction times and the proportion of correct responses.

**Table 2:** Results from Experiment 2, Kanji–Hiragana semantic decision

	Kanji			Hiragana		
	*M*	*SD*	%Acc	*M*	*SD*	*%*Acc
*Related*						
Non-switch	641	137	95	650	139	95
Switch	656	129	94	657	131	94
Switch cost =	15			7		
*Unrelated*						
Non-switch	692	146	95	709	178	93
Switch	704	158	94	713	183	97
Switch cost =	12			4		

Mean response times (in milliseconds), standard deviations, as well as accuracy rates (%Acc), for Kanji and Hiragana words at each trial type. Switch-cost = Switch trials minus non-switch trials

The data were analysed using 2 × 2 related ANOVA (for both participants and items) with the factors of script (Kanji vs. Hiragana) and switch (non-switch vs. switch) separately for the semantically related and unrelated trials. The ANOVAs for the related trials revealed no main effect of script, *Fs* < 1, the main effect of switch was only significant by items, *F*_1_(1, 27) = 2.89, *MSe* = 1028, *p* = .101, η_p_^2^ = .10; *F*_2_(1, 79) = 5.20, *MSe* = 2715, *p* = .025, η_p_^2^ = .06. The script by switch interaction was non-significant, *Fs* < 1. A similar analysis conducted for the unrelated trials revealed no main effect of script, *F*_1_ < 1; *F*_2_(1, 79) = 2.46, *MSe* = 5026, *p* = .120, η_p_^2^ = .03; switch, *F*_1_(1, 27) = 1.84, *MSe* = 997, *p* = .186, η_p_^2^ = .06; *F*_2_(1, 79) = 2.01, *MSe* = 3079, *p* = .160, η_p_^2^ = .03; or switch by script interaction, *Fs* < 1.

The accuracy rate was very high for all conditions (all > 92%). Two-way ANOVAs on the accuracy data of the semantically related trials found a significant main effect of switch by participants, *F*_1_(1, 27) = 6.46, *MSe* = 001, *p* = .017, η_p_^2^ = .19; *F*_2_ < 1. Two consecutively presented words were judged as “related” more accurately when the two words were written in the same script than in different scripts (95 vs. 93%). The main effect of script, *F*_1_ < 1; *F*_2_(1, 79) = 2.03, *MSe* = 011, *p* = .158, η_p_^2^ = .03; and the script by switch interaction were non-significant, *Fs* < 1.

An equivalent analysis for the semantically unrelated trials revealed a significant main effect of switch by participants only, *F*_1_(1, 27) = 7.54, *MSe* = .001, *p* = .011, η_p_^2^ = .22; *F*_2_ < 1, but no effect of script, *F*_1_ < 1; *F*_2_(1, 79) = 2.05, *MSe* = .011, *p* = .156, η_p_^2^ = .03. The script by switch interaction was significant by participants, *F*_1_(1, 27) = 13.43, *MSe* = .001, *p* = .001, *F*_2_ < 1. An examination of the simple main effects showed that the effect was significant only for Hiragana words, revealing that Hiragana switch trials were responded to more accurately than Hiragana non-switch trials. This means that two consecutively presented words were judged as “unrelated” more accurately when the first word was in Kanji and the next word switched to Hiragana, compared to when the two words were both written in Hiragana (97 vs. 93%), *p* < .001. The simple main effect of script was found for both types of trials, showing that Kanji non-switch trials were responded to more accurately than Hiragana non-switch trials (94.6 vs. 93%, *p* = .033). On the contrary, Hiragana switch trials were judged to be unrelated more accurately than Kanji switch trials (96.5 vs. 94.4%, *p* = .024).

When participants are no longer required to read the Kanji words aloud, the finding of slower RTs for Kanji words compared to Hiragana words disappears. This supports the account that reading Kanji words is generally more effortful than reading Hiragana (e.g., Verdonschot et al., 2011, 2013). The results for the related trials found some evidence for switch costs and this time the trend was identical to the findings of Shafiullah and Monsell’s (1999). The magnitude of the cost was also equivalent for the Kanji to Hiragana switch and for the Hiragana to Kanji switch, indicating that there was no familiarity effect. The analysis of the accuracy data replicated this as well. The reaction time for the semantically unrelated trials did not reveal any switch effect, however. Furthermore, the accuracy was higher for the switch than non-switch trials, particularly for Hiragana words. This is likely to be a methodological artefact stemming from asking participants to answer whether the two words are related or unrelated. The response times for unrelated trials were much longer than those for related trials, which suggests that the former was more difficult. To aid the difficult trials, participants might have utilized the visual distinctions of the two scripts as a cue for “unrelated” responses. This might have reduced the response time for unrelated switch trials just to eliminate otherwise visible switch cost. A more detailed interpretation is possible for the superior accuracy of the unrelated Hiragana switch trials over the unrelated Hiragana non-switch trials. Participants were more accurate to decide that the second Hiragana word was unrelated to the first word when the first word was in Kanji compared to when it was in Hiragana. This must reflect Kanji’s advantage of semantic transparency over Hiragana. The equivalent response time for the switch and non-switch conditions indicates that the processing of the second word took place similarly for the two conditions, and this suggests that the difficulty for the non-switch trials arose at the processing of the first word. It might be the case that the semantic processing of the first Hiragana word was not as sufficient as a Kanji word, and that this affected judgement. This result was, however, observed only in the by-participant analysis and is therefore not very robust. This is rather surprising because the Hiragana unrelated condition is the only case which reflects the benefit of quicker semantic processing for Kanji, and this likely indicates that Kanji’s advantage was not as great as initially anticipated and only manifested in possibly the most difficult condition in the present experiment.

In contrast to the initial prediction that this task is completed more quickly for Kanji than Hiragana, no effect of script was found in any condition of the present experiment. This means that the access to semantics was *not* quicker for Kanji, which further highlights the importance of semantic processing for Hiragana reading found in Experiment 1. Again, the current experiment suggests that Hiragana is not processed purely in a phonological manner, and the switch cost might be manifested at an earlier stage, for example during the visual processing of the words, when switching between Kanji and Hiragana.

## Experiment 3: Katakana-Hiragana reading

The purpose of Experiment 3 was to investigate switching between reading Katakana and Hiragana, as well as further explore (and replicate) the involvement of semantics when reading Hiragana. The setup in this experiment was practically identical to Experiment 1, with the only difference that participants were asked to read words written in Katakana and Hiragana. In contrast to Experiment 1, where the effects could be influenced by the differences in the properties of Kanji as a logographic script on the one hand, and Hiragana as a phonological script on the other, the current experiment switches between two highly transparent and phonological scripts. While the Katakana words used in this experiment were all real words (as opposed to transcriptions of words typically written in Kanji) and as such were familiar in their Katakana forms, the Hiragana words were transcriptions of the Katakana words and thus, less familiar. Since these two scripts are visually similar and supposed to use similar reading pathways, the script familiarity is the most likely cause for any observed switch cost. This leads to the prediction that switching from Katakana to Hiragana (from familiar to unfamiliar) is slower than switching from Hiragana to Katakana (from unfamiliar to familiar), in addition to the general pattern of the switch trials being slower than the non-switch trials.

Our previous two experiments, however, questioned the assumption that both Kana scripts use the phonology-to-semantic pathway and suggested that Hiragana processing involves a similar extent of semantic processing as Kanji processing. This may be due to the function of Hiragana which needs to deal with a large number of homophones. As mentioned, the processing of real Katakana words is not subjected to the homophone problem as frequently (being foreign loanwords, and by nature, containing fewer homophones), and thus the Katakana reading pathway may follow a phonology-to-semantic manner more strictly than Hiragana. According to this argument, the Hiragana trials will be influenced by the semantic relatedness of the word pairs more strongly than the Katakana trials. Regarding the magnitude of the switch cost, switching between different pathways should produce equivalent amount of costs for both switching directions (Shafiulla & Monsell, 1999). So, if Katakana and Hiragana engage in distinguishably different pathways, and the effect is not due to the familiarity of the word in that particular form, then switching from Katakana to Hiragana should be equally difficult as switching from Hiragana to Katakana. Conversely, if Katakana and Hiragana use the same pathways, no switch cost in any condition is expected.

### Method

#### Participants

Twenty-eight native Japanese speakers (20 women and 8 men) living in Japan participated in Experiment 3. Their mean age was 19.7 years (SD = .67 years) and none reported having any reading difficulties. They did not participate in any of the other three experiments reported in this paper.

#### Stimulus materials

Forty semantically related word pairs were created with 80 Katakana words in a similar fashion to the Kanji words used in Experiments 1 and 2. See Tables 7 and 8 of “Appendix” for full lists of the stimuli used. The words had between two and four syllables, and the number of characters in each word varied from three to five. The paired words were phonologically dissimilar to each other. Eight experimental lists as well as eight additional pairs for the practice trials were created in the same manner as in Experiment 1. The font and size of the stimuli used in this experiment were identical to Experiment 1.

#### Procedure

The experimental procedure was identical to Experiment 1.

### Results and discussion

The data were treated in the same way as in Experiment 1 (see Table 3 for the naming times). The proportion of the error responses and responses outside 2.5 SDs were 1.9% of the total number of responses, and these were removed from the analysis.

**Table 3:** Results from Experiment 3, Katakana–Hiragana reading

	Katakana		Hiragana	
	*M*	*SD*	*M*	*SD*
*Related*				
Non-switch	563	71	561	73
Switch	572	77	573	75
Switch cost =	9		12	
*Unrelated*				
Non-switch	562	72	575	75
Switch	577	75	565	75
Switch cost =	15		− 10	

Mean response times (in milliseconds) and standard deviations, for Katakana and Hiragana words at each trial type. Switch-cost = Switch trials minus non-switch trials

The correct naming latencies were analysed by 2 × 2 × 2 related ANOVAs (by participants and items separately), with the factors of relatedness (semantically related vs. unrelated), script (Katakana vs. Hiragana) and switch (switch vs. non-switch). The analysis revealed the significant main effect of switch, *F*_1_(1, 27) = 13.10, *MSe* = 177, *p* = .001, η_p_^2^ = .33; *F*_2_(1, 79) = 11.48, *MSe* = 739, *p* = .001, η_p_^2^ = .13, with shorter RTs for non-switch than switch trials (565 vs. 572 ms). There was neither a main effect of relatedness, *F*_1_(1, 27) = 2.30, *MSe* = 168, *p* = .141, η_p_^2^ = .08; *F*_2_ < 1, nor of script, *Fs* < 1. The relatedness by script interaction was non-significant both across participants and items, *Fs* < 1. The presence of the two-way interaction between relatedness and switch, *F*_1_(1, 27) = 5.19, *MSe* = 147, *p* = .031, η_p_^2^ = .16; *F*_2_(1, 78) = 2.24, *MSe* = 705, *p* = .138, η_p_^2^ = .03, as well as script by switch, *F*_1_(1, 27) = 13.67, *MSe* = 128, *p* = .001, η_p_^2^ = .34; *F*_2_(1, 78) = 2.51, *MSe* = 1158, *p* = .117, η_p_^2^ = .03, are inconclusive due to the inconsistent findings between the by participants and by item analyses, but these are clearly modified by a significant three-way interaction found for both analyses, *F*_1_(1, 27) = 25.84, *MSe* = 110, *p* < .001, η_p_^2^ = .49; *F*_2_(1, 78) = 11.72, *MSe* = 921, *p* = .001, η_p_^2^ = .13.

To break down the three-way interaction, 2 × 2 (relatedness: related vs. unrelated; switch: switch vs. non-switch) ANOVAs were carried out separately for Katakana and Hiragana. For Katakana, the analysis found a significant main effect of switch, *F*_1_(1, 27) = 25.46, *MSe* = 159, *p* < .001, η_p_^2^ = .49; *F*_2_(1, 79) = 11.72, *MSe* = 1049, *p* = .001, η_p_^2^ = .13, revealing the presence of a switch cost signified by faster RTs for non-switch than switch trials (562 vs. 574 ms). The main effect of relatedness, *F*_1_(1, 27) = 1.92, *MSe* = 86, *p* = .117, η_p_^2^ = .07; *F*_2_ < 1, and the relatedness by switch interaction, *F*_1_(1, 27) = 3.30, *MSe* = 100, *p* = .080, η_p_^2^ = .11; *F*_2_(1, 79) = 2.05, *MSe* = 832, *p* = .156, η_p_^2^ = .03, were both non-significant. For Hiragana, neither the main effect of relatedness, *F*_1_(1, 27) = 1.40, *MSe* = 160, *p* = .247, *F*_2_ < 1, nor the main effect of switch, *Fs* < 1, were significant, but the relatedness by switch interaction was, *F*_1_(1, 27) = 20.84, *MSe* = 157, *p* < .001, η_p_^2^ = .44; *F*_2_(1, 78) = 12.87, *MSe* = 802, *p* = .001, η_p_^2^ = .14. Simple main effects showed a switch cost for the related conditions (573 vs. 561 ms, *p* = .003) while for the unrelated condition, switch trials were *faster* than non-switch trials (565 vs. 575 ms, *p* = .044). Additionally, for the non-switch trials, the RTs for the related trials were faster than for the unrelated trials (561 vs. 575 ms, *p* < .001), whereas unrelated trials were slightly faster than related trials for switch-trials (565 vs. 573 ms, *p* = .044). A three-way analysis of variance of the proportions of errors and excluded responses showed no significant results.

For Katakana trials, there was a general switch cost, indicating that Katakana and Hiragana are clearly distinct, but there was no effect of semantic relatedness. The absence of semantic facilitation supports the argument that Katakana can be processed in a more direct phonological fashion. No main effect of relatedness in the three-way ANOVA for this experiment implies that the role of semantic processing is somewhat more redundant for Kana reading compared to Kanji reading (a main effect of relatedness was found in Experiment 1). However, semantic relatedness had some impact for Hiragana reading, and the Hiragana trials showed a switch cost only for semantically related trials. The unrelated Hiragana non-switch trials were *slower* than the unrelated switch trials. A switch cost caused by word familiarity would be demonstrated by relatively slow responses for Hiragana switch trials, that is, slower to read a Hiragana word that followed a Katakana word than that followed a Hiragana word. The present results, however, showed the opposite pattern, especially in the unrelated condition. Therefore, the cost is likely to signify the switching of two different reading processes rather than a familiarity effect.

It is an interesting finding that the responses for unrelated Hiragana switch trials appear to be aided by the previously presented Katakana word while this was an impediment for related trials. It was initially anticipated that the Hiragana unrelated switch condition was the hardest because firstly, participants have to switch between scripts, secondly they have to read out the word written in its unfamiliar form, and finally the two words were unrelated. A number of different effects may simultaneously be at play here. For the unrelated conditions, we found that the trials where the second word was preceded by the familiar script Katakana were faster than the trials where the second word was preceded by a Hiragana word. If the effect were dependent on the familiarity of the second word, we would expect faster naming times for trials where the second word in a pair was written in Katakana (irrespective of the script of the first word). If the effect, instead, were due to script switching alone, we would expect faster reaction times for the non-switch than switch trials. If the effect of familiarity and the effect of script switching are approximately equal in magnitude, we would expect faster naming times for Katakana–Katakana than Hiragana–Hiragana trials, and faster naming times for Hiragana–Katakana than Katakana–Hiragana trials. However, the observed results differs from these predictions with regards to the Katakana–Hiragana trials, which are nearly as fast as the Katakana–Katakana trials. One possible explanation is the existence of a “carry-over” effect, whereby the speediness of naming the first word carries over to facilitate the second word. If we assume that this carry-over effect is at play, and combine it with the effects of familiarity and script switching, we might end up with results in line with those found in Experiment 3. These effects, coupled with the semantic relatedness manipulation, are difficult to detangle in the current study, but will need to be systematically studied. For now, our findings suggest that semantic processing is important for Hiragana reading. The present results, therefore, indicate that the reading pathways for Katakana and Hiragana are clearly distinct and this was manifested as the robust switch cost found for Katakana reading.

## Experiment 4: Katakana-Hiragana semantic decision

Finally, Experiment 4 attempted to further examine the degree of semantic involvement when switching between processing of the two phonologically transparent scripts Katakana and Hiragana when participants were not required to read the words aloud. Experiment 4 used the same design as Experiment 2, with the stimuli from Experiment 3. This semantic decision task requires a deeper semantic processing than the automated process of reading. An interesting question here is whether lexical representations of Japanese words are script specific. If so, the familiar Katakana form should gain quicker activation of the lexical representations and thus we will observe generally faster responses for Katakana trials (manifesting in a main effect of script in the analysis). Also, this connects to the script familiarity effect, which expect slower responses for the Hiragana switch trials compared to the Katakana switch trials. Our previous experiments, however, have not found an effect of familiarity and thus an absence of the main effect of script would confirm consistency across experiments.

### Method

#### Participants

Twenty-eight native Japanese speakers (21 women and 7 men) living in Japan participated. Their mean age was 19.4 years (SD = .92 years) and none of the participants reported having any reading difficulties. They did not participate in any other experiments in the present research.

#### Stimulus materials

The materials used in this experiment were identical to Experiment 3.

#### Procedure

The experimental procedure was identical to Experiment 2.

### Results and discussion

The data were treated in the same way as in Experiment 2 (see Table 4 for naming times) and correspondingly, the reaction times for the correct responses were analysed by 2 × 2 related ANOVAs with the factors of script (Katakana vs. Hiragana) and switch (switch vs. non-switch) conducted separately for the semantically related and unrelated trials.

**Table 4:** Results from Experiment 4, Katakana–Hiragana semantic decision

	Katakana			Hiragana		
	*M*	*SD*	%Acc	*M*	*SD*	*%*Acc
*Related*						
Non-switch	690	137	95	718	134	94
Switch	741	150	94	708	150	93
Switch cost =	51			− 10		
*Unrelated*						
Non-switch	740	156	97	745	152	98
Switch	733	135	98	725	148	99
Switch cost =	− 7			− 20		

Mean response times (in milliseconds), standard deviations, as well as accuracy rates (%Acc), for Kanji and Hiragana words at each trial type. Switch-cost = Switch trials minus non-switch trials

The analysis of the RTs for the related trials revealed a significant main effect of switch, *F*_1_(1, 27) = 6.10, *MSe* = 2027, *p* = .020, η_p_^2^ = .18; *F*_2_(1, 79) = 17.15, *MSe* = 2823, *p* < .001, η_p_^2^ = .18; RTs were faster for non-switch than switch trials (704 vs. 725 ms). The main effect of script was absent, *Fs* < 1, but a significant switch by script interaction was found, *F*_1_(1, 27) = 27.26, *MSe* = 951, *p* < .001, η_p_^2^ = .50; *F*_2_(1, 79) = 17.86, *MSe* = 3591, *p* < .001, η_p_^2^ = .18. An examination of the simple main effects of script for the interaction showed that a correct “related” decision was made more quickly for Katakana words than for Hiragana words when there was no script switch for the two consecutively presented words (690 vs. 718 ms, *p* = .003). However, the reverse trend was found for the switch trials, with faster RTs for Hiragana than Katakana switch trials (708 vs. 741 ms, *p* = .002). A strong simple main effect of switch was observed for Katakana words, showing faster responses for the non-switch trials over the switch trials, (690 vs. 741 ms, *p* < .001) but this effect was absent for Hiragana words (*p* = .415). A similar analysis conducted for the unrelated trials revealed no main effect of script, *Fs* < 1, or switch, *F*_1_(1, 27) = 3.31, *MSe* = 1540, *p* = .080, η_p_^2^ = .11; *F*_2_(1, 79) = 3.34, *MSe* = 4586, *p* = .072, η_p_^2^ = .04. The script by switch interaction was also non-significant, *Fs* < 1.

The accuracy rates were very high for all conditions (all > 92%). A two-way analysis of variance on the accuracy data of the semantically related trials did not find any effects that were consistently significant across both participants and items.

Overall, the response times were equivalent for the two Kana scripts indicating that words written in a more familiar form did not allow faster activation of their lexical representations. A robust switch cost for Katakana was found for the related condition. A comparison of the switch trials revealed that switching from Hiragana to Katakana (unfamiliar to familiar) took longer than switching from Katakana to Hiragana (familiar to unfamiliar), and thus there is no evidence of a script familiarity effect. Consistent with the Kanji semantic decision task, no switch cost was observed for the unrelated conditions. Easiness to judge semantically unrelated pairs in different script as “unrelated” might have eliminated the switch costs. It is puzzling that the cost was not observed for the switch from Katakana to Hiragana in the related condition since it was present for the reading experiment and the similar cost was found for the switch from Kanji to Hiragana in Experiment 2. Although statistically non-significant, the responses for Hiragana switch trials were slightly faster and more accurate than non-switch trials for both related and unrelated conditions. This is similar to the accuracy result for the Hiragana unrelated condition in Experiment 2, showing that when the first word of the pair was in Kanji, the response for the second Hiragana word was more accurate than when both words were in Hiragana. Where reaction times are concerned, this trend for the current experiment was also bigger for the unrelated condition, and a similar pattern was observed even for the unrelated Hiragana condition in Experiment 3. This suggest that seeing the first word written in familiar form somehow aids the response for the second Hiragana word when the pair was unrelated. However, most of these results were not statistically robust, and so further investigations are necessary. Nonetheless, the robust cost for the Hiragana to Katakana switch indicates that the processing styles for the two Kana scripts are more dissimilar than initially anticipated.

## General discussion

The present study aimed to investigate the underlying mechanism of script switch effects in Japanese. Across four experiments, participants either read aloud or made a semantic decision when presented with pairs of words that were written either in the same orthography (within-script), or two different Japanese orthographies (cross-script). First of all, the familiarity of the script did not influence the magnitude of the switch cost, and this was true for both the Kanji–Hiragana and Katakana–Hiragana switch experiments. Measuring the pure extent of switch cost was found to be difficult for Kanji–Hiragana switch due to the general difficulty in reading aloud Kanji words. Although small, the switch cost found in Experiment 2 suggests that there is a fixed amount of cost involved in switching between Kanji and Hiragana, but the mechanism cannot be narrowed down to the switch of two separate pathways (semantics-to-phonology for Kanji and phonology-to-semantics for Hiragana). Furthermore, the present results consistently showed that Hiragana is not processed purely in a phonology-to-semantics manner and that some level of semantic processing is likely to be crucial for Hiragana reading. In contrast, Katakana reading reflects a phonology-to-semantics route much more clearly, indicating that the processing styles for the two types of Kana are distinct. Indeed, the function of Hiragana greatly differs from that of Katakana and is used to write complete words, be of grammatical aid (function words), and be combined with morphological Kanji characters to, for example, conjugate verbs. This multifunctional nature of Hiragana leads it to deal with a large number of homophones and near-homophones whose pronunciations depend on their meaning, something that is not an issue for Katakana words. The script switch costs demonstrated by the Japanese readers for these two Kana orthographies also support the notion that Hiragana and Katakana are not fully interchangeable. Precisely how Hiragana is processed remains to be investigated more clearly in future studies. For example, it is not clear whether Hiragana words with no possible homophones are regarded as having the similar extent of semantic transparency as Kanji or Katakana words. For now, we demonstrate that it is not likely to be processed in a strict phonology-to-semantics fashion.

The present research supports Shafiullah and Monsell’s (1999) conclusion that the script familiarity is not the cause of the script switch. At the same time, however, our findings contradict others which have observed script familiarity effects (e.g., Besner & Hildebrandt, 1987; Chikamatsu, 2006; Tamaoka, 1997). Although almost all words presented to the participants in the present study were either written in Kanji or Katakana in their familiar forms, reading them aloud when they were written in Hiragana was as fast as when they were written in familiar scripts. Indeed, participants were *faster* at reading the Hiragana transcriptions of Kanji words compared to reading the actual Kanji words. This indicates that Hiragana reading is generally fast and relatively effortless, especially compared to Kanji reading. This may reflect the multifunctional nature of Hiragana (which is used in many different contexts), as well as the nature of Kanji reading (which is generally more effortful). We would also like to point out that the experiments which have typically found a script familiarity effect have used considerably different methodologies. For example, Besner and Hildebrandt (1987) found faster processing times for real Katakana words (Katakana as a familiar script) compared with both Katakana transcriptions of Kanji words and nonwords written in Katakana (Katakana as an unfamiliar script). Therefore, their finding, while significant, may not be transferable to our stimuli, where the familiar and unfamiliar scripts were two distinct orthographies, which are evidently not as transposable as previously assumed. Finally, the issue of familiarity could be broken down into two levels, namely the word versus the character level. While a single word can be more familiar when written in Katakana, single Hiragana characters will, on a whole, be more familiar than Katakana. These two types of familiarity (specific word vs. single characters of the more familiar script) may be able to account for the discrepancy of familiarity effect between different studies. The interaction between these two familiarity levels, however, remains an empirical question for now.

The present research also revealed some intriguing characteristics of Kanji. Kanji reading was found to be much more effortful than Kana reading, and most interestingly, it was found that reading a Kanji word when having just read another Kanji word is slower than when reading a Kanji word following a Hiragana transcription (for which there presumably is no stored lexical representation). This is counter-intuitive, especially considering that the Kanji words used in the current study were all highly common and “easy” words. One possible reason for this is that reading multiple Kanji words in a consecutive fashion was an unusual event for the Japanese reader. This is because the multiple orthographies of Japanese are mixed and combined in everyday use, and consequently, native readers become accustomed to regularly switching between reading words in the different scripts, within a single sentence, and sometimes even within a single word, with Kanji and Hiragana frequently being combined to form a single word. Even in instances where the entire content word is comprised of Kanji characters, it is usually followed by a function word written in Hiragana, and so Japanese readers may instinctively expect to see a switch of orthographies, particularly when encountering a Kanji word. Consequently, Kanji non-switch trials were slower than both Kanji switch and Hiragana switch trials. Indeed, some studies have found that Kanji-Kana mixed sentences are read faster than sentences comprised of only Kana (e.g., Kitao, 1960; Sakamoto & Makita, 1973). Importantly though, those studies measured word recognition in sentence reading, and this is very different from the present experiments which showed each word individually with no contextual clues. Considering that most Japanese common words are written in Kanji rather than in Hiragana, it can be said that reading consecutive Hiragana words is more unnatural than reading multiple Kanji words, although Hiragana reading would not have suffered greatly from this unusualness since the script is phonologically transparent. Therefore, it is not very likely that solely the unnaturalness of the experimental condition slowed down the reading time for a Kanji word following another Kanji word. Another possibility is that reading a Kanji word slows down reading of subsequent words, irrespective of whether the subsequent word is in Kanji or Hiragana. However, this explanation seems unlikely, as script switch costs were found in Experiment 3, where none of the stimuli were in Kanji. Also, Kanji non-switch trials were not slower than other trials in the semantic decision task, indicating that reading separate Kanji words *aloud* entails special difficulty.

One explanation for the difficulty in reading consecutive Kanji words is brought by the characteristics of this script, which often have multiple pronunciations, *On*- and *Kun*-*readings*. These readings are often regulated depending on the context of the Kanji word, following a set of rules that all Japanese readers are taught to follow. For example, the Kanji for ‘car’ is pronounced “kuruma” and written using the following Kanji: 車. However, when the same Kanji character is written as part of the two-compound Kanji word電車 (‘train’, pronounced as “densha”), the second Kanji character is now pronounced as “sha”. Because Kanji words are often compound words, it is plausible that reading a Kanji character affects subsequent processing of Kanji words because the reader is, generally speaking, accustomed to holding off the final decision of which pronunciation to choose until all relevant information is processed. This would explain why the non-switch trials, where participants read two Kanji words in succession, were read slower than the switch trials when participants first read a Hiragana word and then a Kanji word. We purposefully avoided these types of pairings for obvious reasons, and so this argument is only speculation for now and needs to be investigated more thoroughly in future research. However, this could provide an explanation for the general processes involved in reading Japanese Kanji. Future research will also need to more closely investigate precisely why the general difficulty of reading aloud Kanji words diminishes the semantic priming effect. If we assume that reading Kanji aloud uses the meaning-to-phonology pathway and that the general difficulty in naming Kanji mainly involves phonological processing, it remains unclear how the phonological process hinders the preceding semantic processing. One possibility is that the phonological and the semantic processes start in parallel and compete with each other, leading the phonological process to disturb the competing semantic process thereby suppressing the semantic relatedness effects.

Finally, we address some methodological considerations for the current experiments and possible improvements. Selecting items often poses difficulties in the field of psycholinguistic research and the current study is not an exception. The words used in the present study lacks manipulations in various indices such as familiarity, frequency, and imageability. Importantly, though, we mainly focused on the results which were present for both by item and by participant analyses. Shafiullah and Monsell (1999) also examined and concluded that the script switch cost is relatively independent from a number of within-script characteristics. The repetition of the same word in the experiment is another concern, but it is extremely difficult to avoid repetition, and Shafiullah and Monsell’s (1999) careful examination of this issue revealed no impact on the switch cost. In addition to this, the unnaturalness of reading consecutive Kanji words may need to be modified in some ways, for example by creating a situation in which participants need to read a list of Kanji words, such as a shopping list, to avoid them reading a word presented one by one on the screen. Our semantic decision task also suffered some issues due to the response requirement. In this case, asking participants to classify each word in some semantic categories (e.g., colour or shape) may be more preferable than asking them to identify the relationship between the paired words. However, we would like to point out that despite the variation in the stimuli used with regards to characteristics such as familiarity and imageability, the results are consistent and robust across the experiments reported.

To conclude, the three scripts investigated in the current research have distinct functions and their processing have some similarities and differences. The script switch cost is clearly present. Even if the mechanism is still not fully clear, the familiarity effect can most likely be dismissed. The present research suggests that the distinction between the semantic-to-phonology and the phonology-to-semantic pathways may be more flexible and dependent on the function of the word than initially anticipated. The present research has successfully demonstrated the importance of semantic processing in reading Hiragana, and raised some interesting points regarding the effortful processing involved in reading aloud Kanji words, which needs to be investigated more closely in future studies.

### Electronic supplementary material

Below is the link to the electronic supplementary material.

Supplementary material 1 (DOCX 17 kb)

## Appendix: Word lists

See Tables 5, 6, 7, and 8.

**Table 5:** Kanji–Hiragana related word pairs

	Kanji		Hiragana		Phonetics		Meaning	
1	夏	冬	なつ	ふゆ	natsu	fuyu	summer	winter
2	犬	猫	いぬ	ねこ	inu	neko	dog	cat
3	村	町	むら	まち	mura	machi	village	town
4	竹	松	たけ	まつ	take	matsu	bamboo	pine
5	土	泥	つち	どろ	tsuchi	doro	soil	mud
6	父	母	ちち	はは	chichi	haha	father	mother
7	山	海	やま	うみ	yama	umi	mountain	sea
8	床	壁	ゆか	かべ	yuka	kabe	floor	wall
9	万	億	まん	おく	man	oku	ten thousands	100 million
10	朝	夜	あさ	よる	asa	yoru	morning	night
11	星	月	ほし	つき	hoshi	tsuki	star	moon
12	雨	風	あめ	かぜ	ame	kaze	rain	wind
13	米	麦	こめ	むぎ	kome	mugi	rice	wheat
14	男	女	おとこ	おんな	otoko	onna	male	female
15	家	庭	いえ	にわ	ie	niwa	house	garden
16	空	雲	そら	くも	sora	kumo	sky	cloud
17	森	川	もり	かわ	mori	kawa	forest	river
18	横	縦	よこ	たて	yoko	tate	width	lengthwise
19	花	種	はな	たね	hana	tane	flower	seeds
20	心	体	こころ	からだ	kokoro	karada	mind	body
21	大学	高校	だいがく	こうこう	daigaku	koukou	university	high school
22	入学	卒業	にゅうがく	そつぎょう	nyuugaku	sotsugyou	enter a school	graduating
23	大人	子供	おとな	こども	otona	kodomo	adult	child
24	天使	悪魔	てんし	あくま	tennshi	akuma	angel	devil
25	田舎	都会	いなか	とかい	inaka	tokai	rural	urban
26	地球	火星	ちきゅう	かせい	chikyu	kasei	earth	mars
27	教師	生徒	きょうし	せいと	kyoushi	seito	teacher	student
28	平行	垂直	へいこう	すいちょく	heikou	suityoku	parallel	vertical
29	病院	薬局	びょういん	やっきょく	byouinn	yakkyoku	hospital	symbol
30	文字	記号	もじ	きごう	moji	kigou	letter	past
31	未来	過去	みらい	かこ	mirai	kako	future	earthquake
32	火事	地震	かじ	じしん	kaji	jishinn	fire	pharmacy
33	廊下	階段	ろうか	かいだん	rouka	kaidan	corridor	stairs
34	上司	同僚	じょうし	どうりょう	jyoushi	douryou	boss	colleague
35	柔道	空手	じゅうどう	からて	jyuudou	karate	judo	karate
36	野球	水泳	やきゅう	すいえい	yakyuu	suiei	baseball	swimming
37	果物	野菜	くだもの	やさい	kudamono	yasai	fruits	vegetables
38	信号	道路	しんごう	どうろ	shinngou	douro	traffic lights	road
39	寿司	刺身	すし	さしみ	sushi	sashimi	sushi	sashimi
40	部分	全体	ぶぶん	ぜんたい	bubunn	zenntai	parts	whole

**Table 6:** Kanji–Hiragana unrelated word pairs

	Kanji		Hiragana		phonetics		meaning	
1	横	冬	よこ	ふゆ	yoko	fuyu	width	winter
2	竹	猫	たけ	ねこ	take	neko	bamboo	cat
3	雨	町	あめ	まち	ame	machi	rain	town
4	花	万	はな	まん	hana	man	flower	ten thousands
5	夏	泥	なつ	どろ	natsu	doro	summer	mud
6	松	母	まつ	はは	matsu	haha	pine	mother
7	朝	海	あさ	うみ	asa	umi	morning	sea
8	山	壁	やま	かべ	yama	kabe	mountain	wall
9	床	億	ゆか	おく	yuka	oku	floor	100 million
10	星	庭	ほし	にわ	hoshi	niwa	star	garden
11	森	月	もり	つき	mori	tsuki	forest	moon
12	土	風	つち	かぜ	tsuchi	kaze	soil	wind
13	家	麦	いえ	むぎ	i.e.	mugi	house	wheat
14	心	女	こころ	おんな	kokoro	onna	mind	female
15	犬	夜	いぬ	よる	inu	yoru	dog	night
16	米	雲	こめ	くも	kome	kumo	rice	cloud
17	男	体	おとこ	からだ	otoko	karada	male	body
18	空	縦	そら	たて	sora	tate	sky	lengthwise
19	父	種	ちち	たね	chichi	tane	father	seeds
20	村	川	むら	かわ	mura	kawa	village	river
21	未来	高校	みらい	こうこう	mirai	koukou	future	high school
22	野球	天使	やきゅう	てんし	yakyuu	tennshi	baseball	angel
23	信号	子供	しんごう	こども	shinngou	kodomo	traffic lights	child
24	田舎	悪魔	いなか	あくま	inaka	akuma	rural	devil
25	卒業	都会	そつぎょう	とかい	sotsugyou	tokai	graduating	urban
26	廊下	火星	ろうか	かせい	rouka	kasei	corridor	mars
27	地球	生徒	ちきゅう	せいと	chikyu	seito	earth	student
28	平行	地震	へいこう	じしん	heikou	jishinn	parallel	earthquake
29	火事	寿司	かじ	すし	kaji	sushi	fire	sushi
30	大学	記号	だいがく	きごう	daigaku	kigou	university	symbol
31	柔道	薬局	じゅうどう	やっきょく	jyuudou	yakkyoku	judo	pharmacy
32	病院	垂直	びょういん	すいちょく	byouinn	suityoku	hospital	vertical
33	教師	階段	きょうし	かいだん	kyoushi	kaidan	teacher	stairs
34	果物	同僚	くだもの	どうりょう	kudamono	douryou	fruits	colleague
35	入学	空手	にゅうがく	からて	nyuugaku	karate	enter a school	karate
36	野菜	水泳	やさい	すいえい	yasai	suiei	vegetables	swimming
37	文字	過去	もじ	かこ	moji	kako	letter	past
38	大人	道路	おとな	どうろ	otona	douro	adult	road
39	部分	刺身	ぶぶん	さしみ	bubunn	sashimi	parts	sashimi
40	上司	全体	じょうし	ぜんたい	jyoushi	zenntai	boss	whole

**Table 7:** Katakana–Hiragana related word pairs

	Katakana		Hiragana		phonetics		meaning	
1	セーター	シャツ	せーたー	しゃつ	se-ta-	shatu	jumper	shirt
2	ジャム	バター	じゃむ	ばたー	jyamu	bata-	jam	butter
3	パソコン	プリンタ	ぱそこん	ぷりんた	pasokon	purinnta	PC	printer
4	テレビ	ラジオ	てれび	らじお	terebi	rajio	TV	radio
5	キーボード	マウス	きーぼーど	まうす	ki-bo-do	mausu	keyboard	mouse
6	ギター	ピアノ	ぎたー	ぴあの	gita-	piano	guitar	piano
7	ゴールド	シルバー	ごーるど	しるばー	go-rudo	siruba-	gold	silver
8	スピーカー	マイク	すぴーかー	まいく	supi-ka-	maiku	speaker	microphone
9	ナイフ	フォーク	ないふ	ふぉーく	naifu	fo-ku	knife	fork
10	ネクタイ	リボン	えくたい	りぼん	nekutai	ribonn	tie	ribbon
11	レタス	トマト	れたす	とまと	nekutai	tomato	lettuce	tomato
12	テニス	サッカー	てにす	さっかー	tenisu	sakka-	tennis	football
13	スニーカー	ブーツ	すにーかー	ぶーつ	suni-ka-	bu-tsu	trainers	boots
14	ワイン	カクテル	わいん	かくてる	wainn	kakuteru	wine	cocktail
15	レコード	アルバム	れこーど	あるばむ	reko-do	arubamu	record	album
16	スカート	ドレス	すかーと	どれす	suka-to	doresu	skirt	dress
17	キャベツ	ピーマン	きゃべつ	ぴーまん	kyabetsu	pi-mann	cabbage	pepper
18	パンチ	キック	ぱんち	きっく	pannchi	kikku	punch	kick
19	ハイヒール	サンダル	はいひーる	さんだる	haihi-ru	sanndaru	high heels	sandals
20	ペンギン	フラミンゴ	ぱんぎん	ふらみんご	pennginn	furaminngo	penguin	flamingo
21	ブレーキ	アクセル	ぶれーき	あくせる	bure-ki	akuseru	brake	accelerator
22	ディナー	ランチ	でぃなー	らんち	dina-	rannchi	dinner	lunch
23	ソファー	テーブル	そふぁー	てーぶる	sofa-	te-buru	sofa	table
24	カンガルー	コアラ	かんがるー	こあら	kanngaru-	koara	kangaroo	koala
25	バナナ	キウイ	ばなな	きうい	banana	kiui	banana	kiwi
26	キッチン	リビング	きっちん	りびんぐ	kicchinn	libinngu	kitchen	living room
27	パンダ	ゴリラ	ぱんだ	ごりら	pannda	gorira	panda	gorilla
28	シャンプー	リンス	しゃんぷー	りんす	shannpu-	rinnsu	shampoo	conditioner
29	マンゴー	グレープ	まんごー	ぐるーぷ	manngo-	gure-pu	mango	grape
30	ジーンズ	ズボン	じーんず	ずぼん	ji-nzu	zubons	jeans	trousers
31	サラダ	パスタ	さらだ	ぱすた	sarada	pasuta	salad	pasta
32	マッチ	ライター	まっち	らいたー	macchi	raita-	matches	lighter
33	グローブ	バット	ぐろーぶ	ばっと	guro-bu	batto	baseball glove	baseball bat
34	ドラム	ベース	どらむ	べーす	doramu	be-su	drums	bass
35	ハンドル	タイヤ	はんどる	たいや	hanndoru	taiya	steering wheel	tire
36	メール	ファックス	めーる	ふぁっくす	me-ru	fakkusu	mail	fax
37	ジャズ	ロック	じゃず	ろっく	jazu	rokku	jazz	rock
38	ヨーロッパ	アフリカ	よーろっぱ	あふりか	yo-roppa	afurika	Europe	Africa
39	タクシー	パトカー	たくしー	ぱとかー	takushi-	patoka-	taxi	police car
40	エアコン	ヒーター	えあこん	ひーたー	eakonn	hi-ta-	air conditioner	heater

**Table 8:** Katakana–Hiragana unrelated word pairs

	Katakana		Hiragana		phonetics		meaning	
1	ディナー	シャツ	でぃなー	しゃつ	dina-	shatu	dinner	shirt
2	タクシー	マウス	たくしー	まうす	takushi-	mausu	taxi	mouse
3	ジャム	ピアノ	じゃむ	ぴあの	jyamu	piano	jam	piano
4	サラダ	シルバー	さらだ	しるばー	sarada	siruba-	salad	silver
5	テニス	マイク	てにす	まいく	tenisu	maiku	tennis	microphone
6	パソコン	リボン	ぱそこん	りぼん	pasokon	ribonn	PC	ribbon
7	ゴールド	トマト	ごーるど	とまと	go-rudo	tomato	gold	tomato
8	シャンプー	サッカー	しゃんぷー	さっかー	shannpu-	sakka-	shampoo	football
9	スカート	カクテル	すかーと	かくてる	suka-to	kakuteru	skirt	cocktail
10	ハイヒール	アルバム	はいひーる	あるばむ	haihi-ru	arubamu	high heels	album
11	ギター	ドレス	ぎたー	どれす	gita-	doresu	guitar	dress
12	ハンドル	ピーマン	はんどる	ぴーまん	hanndoru	pi-mann	steering wheel	bell pepper
13	レタス	サンダル	れたす	さんだる	nekutai	sanndaru	lettuce	sandals
14	ヨーロッパ	アクセル	よーろっぱ	あくせる	yo-roppa	akuseru	Europe	accelerator
15	テレビ	コアラ	てれび	こあら	terebi	koara	TV	koala
16	マッチ	キウイ	まっち	きうい	macchi	kiui	matches	kiwi
17	キャベツ	リビング	きゃべつ	りびんぐ	kyabetsu	libinngu	cabbage	living room
18	ソファー	ゴリラ	そふぁー	ごりら	sofa-	gorira	sofa	gorilla
19	スピーカー	リンス	すぴーかー	りんす	supi-ka-	rinnsu	speaker	conditioner
20	ブレーキ	ズボン	ぶれーき	ずぼん	bure-ki	zubons	brake	trousers
21	ネクタイ	パスタ	ねくたい	ぱすた	nekutai	pasuta	tie	pasta
22	メール	バット	めーる	ばっと	me-ru	batto	mail	baseball bat
23	バナナ	ベース	ばなな	べーす	banana	be-su	banana	bass
24	カンガルー	タイヤ	かんがるー	たいや	kanngaru-	taiya	kangaroo	tire
25	ライター	ファックス	らいたー	ふぁっくす	raita-	fakkusu	lighter	fax
26	スニーカー	ロック	すにーかー	ろっく	suni-ka-	rokku	trainers	rock
27	ドラム	パトカー	どらむ	ぱとかー	doramu	patoka-	drums	police car
28	ナイフ	パンダ	ないふ	ぱんだ	naifu	pannda	knife	panda
29	ジャズ	プリンタ	じゃず	ぷりんた	jazu	purinnta	jazz	printer
30	レコード	バター	れこーど	ばたー	reko-do	bata-	record	butter
31	キッチン	フラミンゴ	きっちん	ふらみんご	kicchinn	furaminngo	kitchen	flamingo
32	グローブ	エアコン	ぐろーぶ	えあこん	guro-bu	eakonn	baseball glove	air conditioner
33	ランチ	キック	らんち	きっく	rannchi	kikku	lunch	kick
34	パンチ	テーブル	ぱんち	てーぶる	pannchi	te-buru	punch	table
35	ワイン	セーター	わいん	せーたー	wainn	se-ta-	wine	jumper
36	マンゴー	ブーツ	まんごー	ぶーつ	manngo-	bu-tsu	mango	boots
37	ジーンズ	グレープ	じーんず	ぐれーぷ	ji-nzu	gure-pu	jeans	grape
38	キーボード	アフリカ	きーぼーと	あふりか	ki-bo-do	afurika	keyboard	Africa
39	ペンギン	ヒーター	ぺんぎん	ひーたー	pennginn	hi-ta-	penguin	heater
40	フォーク	ラジオ	ふぉーく	らじお	fo-ku	rajio	fork	radio

## Footnotes

1 Although Tamaoka (1997) tested second language learners of Japanese, the results may still be informative of the underlying processes when reading Hiragana and Katakana transcriptions of Kanji words.

2 We thank the reviewer who recommended we use linear mixed effect (LME) analyses rather than the reported ANOVAs. Unfortunately, we could not find a perfectly suitable model due to the design of the experiments: the word variations in the present research cannot be regarded as a completely random factor since the same words appeared both in the related and unrelated conditions being paired with different words. Nonetheless, we ran LME analyses using a justifiable, albeit imperfect model and found generally a similar pattern to the reported ANOVAs. More detailed information about the analyses are available in Supplementary Materials.
