# Supplementary material for: The role of semantic processing in reading Japanese orthographies: an investigation using a script-switch paradigm
Source: Read Writ. 2017 Nov 8;31(3):503–31. doi: 10.1007/s11145-017-9796-3 (PMC5803283; doi:10.1007/s11145-017-9796-3)
Supplement: Supplementary file 1 — Supplementary material 1 (DOCX 17 kb) [file 11145_2017_9796_MOESM1_ESM.docx]

**Supplementary Materials**

**Data Analysis**

The reaction time data from all four experiments and the accuracy data from the semantic decision experiments were additionally analysed using linear mixed effects (LME) models fit by REML. For the analyses the calculations of the p-values were based on the Satterthwaite’s approximation for the degrees of freedom. We used R version 3.3.0 (R Development Core Team, 2008) with *lme4* package (Bates, Maechler, Bolker, & Walker, 2015). For Experiment 1 and 3, we entered fixed effects of semantic relatedness, script and switch, as well as the all possible two- and three-way interactions into the model. As random effects, we had intercepts for participants and items, as well as by-participant and by-item random slopes for the fixed effects. For Experiment 2 and 4 the analyses were performed separately for related and unrelated conditions, leaving script, switch and a two-way interaction of these as fixed effects. In these models, the item factor was the variation of the second word of each word pair. This treatment, however, may be problematic because the experiments used the same word for both the semantically related and unrelated conditions by re-paring them with different words. Also, the order of the paired words was reversed for half of the participants. These experimental designs and procedures might have violated the independence of each item from the fixed effects, and thus we consider this model as inferior to the by-participants and by-items ANOVA analyses reported in the main text, and which were the intended analyses when the experiments were initially designed. Nevertheless, the LME analyses generally found the same pattern of results as in the ANOVAs.

Experiment 1: Kanji-Hiragana Reading

The analysis revealed a main effect of relatedness (beta = -4.99, *SE* = 1.62, *t*(77) = 3.09, *p* < .01) and of script (beta = 12.83, *SE* = 2.31, *t*(32) = 5.55, *p* < .001). Reading time for the second word of the word pair was faster when previously presented word was semantically related to it. Also, the reading time was faster when the paired words were written in the same script than different scripts. Similar to the ANOVA reported in the main text this analysis also revealed a relatedness by script interaction (beta = -9.39, *SE* = 2.31, *t*(59) = 4.06, *p* < .001).

Experiment 2: Kanji-Hiragana Semantic Decision

No effects were found for the reaction times data. The accuracy data showed a significant main effect of switch, but only for the unrelated trials (beta = 0.01, *SE* = 0.003, *t*(27) = 2.17, *p* < .05).

Experiment 3: Katakana-Hiragana Reading

No effects were found when including all three variables (semantic relatedness, script and switch). However, in light of the highly significant three-way interaction in both the by-participants and the by-items ANOVAs, the data were broken down and analysed using linear mixed effects models with semantic relatedness and switch separately for the Katakana and the Hiragana trials. For the Katakana trials, a significant main effect of switch was found, (beta = 5.44, *SE* = 2.17, *t*(53) = 2.50, *p* < .05), where RTs for non-switch were faster than for switch trials.

Experiment 4: Katakana-Hiragana Semantic Decision

The analysis using reaction time for related condition revealed a significant main effect of switch, (beta = 9.94, *SE* = 3.49, *t*(39) = 2.85, *p* < .01), showing the slower response for switch condition. Additionally, a significant script by switch interaction was found, (beta = 12.03, *SE* = 3.90, *t*(72) = 3.08, *p* < .01). There was no significant main effect of script (*p* > .05). Further, no effects were found for the reaction times for the unrelated trials. For the accuracy data only significant effect found was a main effect of switch for the unrelated trials (beta = 0.008, *SE* = 0.003, *t*(4216) = 2.49, *p* < .05).

References

Bates, D., Maechler, M., Bolker, B., & Walker, S. (2015). Fitting linear mixed-effects models using lme4. Journal of Statistical Software, 67, 148. doi:10.18637/jss.v067.i01.

R Development Core Team (2008). R: A language and environment for statistical computing. R Foundation for Statistical Computing, Vienna, Austria. URL: http://www.R- project.org.
